# Detection of methicillin-resistant *Staphylococcus aureus* (MRSA) in clinical samples of patients with external ocular infection

**Published:** 2018-08

**Authors:** Ashkan Faridi, Amir Tavakoli Kareshk, Mehdi Fatahi-Bafghi, Mahsa Ziasistani, Mohammad Reza Kandehkar Ghahraman, Seyedeh Zeinab Seyyed-Yousefi, Noshin Shakeri, Davood Kalantar-Neyestanaki

**Affiliations:** 1 Student Research Committee, School of Medicine, Kerman University of Medical Sciences, Kerman, Iran; 2 Infectious Diseases Research Center, Birjand University of Medical Sciences, Birjand, Iran; 3 Department of Microbiology, Faculty of Medicine, Shahid Sadoughi University of Medical Sciences, Yazd, Iran; 4 Pathology and Stem Cell Research Center, School of Medicine, Kerman University of Medical Sciences, Kerman, Iran; 5 Department of Pathobiology, School of Public Health, Tehran University of Medical Sciences, Tehran, Iran; 6 Shahid Labafi Nejad Hospital, Shahid Beheshti University of Medical Sciences, Tehran, Iran; 7 Department of Microbiology and Virology, School of Medicine, Kerman University of Medical Sciences, Kerman, Iran

**Keywords:** Eye infection, MRSA, Panton–Valentine leukocidin

## Abstract

**Background and Objectives:**

*Staphylococcus aureus* is the main Gram-positive bacteria isolated from patients with ocular infections. Herein, we describe the pattern of antibiotic resistance, presence of resistance genes including *ermA, ermB, ermC, msrA, mecA* and the *pvl* cytotoxin gene in *S. aureus* isolates collected from patients with external ocular infection.

**Materials and Methods:**

In this study, 8 *S. aureus* isolates were collected from 81 patients that suffered from eye damage. Antibacterial susceptibility of isolates was determined using the Kirby-Bauer disk diffusion method. Resistance genes including *ermA, ermB, ermC, msrA, mecA* and the *pvl* virulence gene were detected by PCR method. Staphylococcal cassette chromosome *mec* (SCC*mec*) in MRSA isolates were detected by the multiplex-PCR method.

**Results:**

Three isolates were resistant to cefoxitin which is considered MRSA. The *mecA* gene was identified in MRSA isolates. SCC*mec* type IV and the *pvl* gene were detected in one of the MRSA isolates that was recovered from a diabetic patient.

**Conclusion:**

The emergence of *S. aureus* isolates belonging to SCC*mec* type IV and *pvl* gene among patients with ocular infection is very serious; therefore, identify genetic characterization of MRSA isolates for empirical therapy and infection control is very important.

## INTRODUCTION

*Staphylococcus aureus* causes a wide range of infections including bacteremia as well as pleuropulmonary, skin and soft tissue infections (1, 2). This bacterium is also responsible for many types of ocular infections including keratitis, conjunctivitis, endophthalmitis and blepharitis (2). Some virulence factors such as Panton-valentine leukocidin (PVL) as well as antibiotic resistance have an important role in increasing tissue damage and failure in antibiotic therapy, respectively (1–3). There are many reports about ocular infection due to methicillin-resistant *S. aureus* (MRSA) isolates (2). In the past decade, infections by MRSA strains have increased in hospital settings and communities (2). MRSA isolates are commonly resistant to a wide range of antibiotics such as aminoglycosides, erythromycin, tetracycline and fluoroquinolones (1–3). The *mecA* gene is on the staphylococcal cassette chromosome *mec* (SCC*mec*) genetic element that causes resistance to β-lactam antibiotics (3–5). SCC*mec* type IV usually is related to community-associated-MRSA (CA-MRSA) and other SCC*mec* types including SCC*mec* types I, II and III related to hospital-acquired MRSA (HA-MRSA) isolates (5, 6). The PVL is a cytotoxin virulence factor produced by some *S. aureus* commonly related to CA-MRSA isolates and causes cell destruction and tissue necrosis (6). Antibiotics such as erythromycin and tetracycline were used for treatment of ocular infections by *S. aureus* (1). Activity of efflux pumps such as *msr* (A/B) and methylation of the ribosomal drug binding site by methylase enzymes encoded by *erm* (A, B, C) genes are important resistance mechanisms to macrolide antibiotics (3, 7). Herein, we described antibiotic susceptibility pattern, persence of *msrA/B, ermA, ermB, ermC, mecA, SCCmec* type and the *pvl* genes among *S. aureus* isolates recovered from patients with external ocular infection in Tehran, Iran.

## MATERIALS AND METHODS

### Bacterial isolates.

In a cross-sectional study, from April 2016 to September 2016, 8 *S. aureus* isolates were collected from clinical samples of 81 different patients suffering from eye damage and purulent discharge admitted to Shahid Labafi Nejad Hospital in Tehran, Iran. Bacterial isolates were considered as *S. aureus* using standard microbiological tests including Gram staining, catalase reaction, coagulase production, β-hemolysis on blood agar base enriched with 5% sheep blood, DNase production and mannitol fermentation on mannitol salt agar media (6). All culture media used in this investigation were supplied by HiMedia, Co, India. Finally, isolates confirmed by amplification of the *nuc* gene by the PCR technique as described previously (6).

### Antibacterial susceptibility tests.

Antibacterial susceptibility of isolates to amikacin (30 μg), ciprofloxacin (5 μg), clindamycin (2 μg), erythromycin (15 μg), gentamicin (10 μg), linezolid (30 μg), tetracycline (30 μg) and trimethoprim/sulfamethoxazole (1.25/23.75 μg) were determined using Kirby-Bauer disk diffusion method on Mueller hinton agar media (CONDA, Co, Spain) (8). Brain hearth infusion agar (BHI; Difco, USA) containing 6 μg/mL vancomycin was used for detection of vancomycin resistant isolates (8). MRSA and inducible clindamycin resistance phenotypes were determined using the CLSI recommendations (8). The *S. aureus* ATCC 29213 was used as the control strain in antibacterial susceptibility tests and the phenotypic method.

### Total DNA extraction.

Total DNA of MRSA isolates were extracted by the boiling method described by Zhang et al. (9).

### Detection of resistance and the *pvl* gene by PCR.

PCR amplification of the *msrA/B, ermA, ermB, ermC, mecA* and *pvl* genes were done with primers listed in Table 1 as described previously (3, 4, 6, 10–13). The PCR products were observed by electrophoresis on 1% agarose gels (SinaClon, Co, Iran). This was followed by DNA Green Viewer (Pars Tous Biotechnology, Co, Iran) staining and analysis.

**Table 1.** The list of oligonucleotide primers were used in this study.

**Targetgene**	**Primer sequence (5′–3′)**	**Annealing temperature (°C)**	**Product size (bp)**	**Reference**
*nuc*	F-GCGATTGATGGTGATACGGTT	60	279	(6)
	R-AGCCAAGCCTTGACGAACTAAAGC			
*mecA*	F-TCCAGATTACAACTTCACCAGG	56	162	(4)
	R-CCACTTCATATCTTGTAACG			
*ermA*	F-TATCTTATCGTTGAGAAGGGATT	56.5	139	(10)
	R-CTACACTTGGCTTAGGATGAAA			
*ermB*	F-CTATCTGATTGTTGAAGAAGGATT	55.5	142	(3)
	R-GTTTACTCTTGGTTTAGGATGAAA			
*ermC*	F-AATCGTCAATTCCTGCATGT	55.5	297	(11)
	R-TAATCGTGGAATACGGGTTTG			
*msrA*	F-GCAAATGGTGTAGGTAAGACAACT	56.5	402	(12)
	R-ATCATCATGTGATGTAAACAAAAT			
*pvl*	F-ATCATTAGGTAAAATGTCTGGACATGATCCA	55	433	(13)
	R-GCATCAASTGTATTGGATAGCAAAAGC			

### Detection of SCC*mec* types by multiplex PCR.

Two different multiplex PCR methods, described by Oliveira et al. and Boye et al., were used for SCC*mec* typing of MRSA isolates (4, 5).

## RESULTS

Of the 81 clinical samples collected from different patients, 8 isolates were identified as *S. aureus* using standard microbiological tests and amplification of the *nuc* gene. Of the 8 *S. aureus*, two isolates were from the surgical external ocular infection of two males (43 and 82-years old) with diabetes mellitus. One isolate was from the surgical external ocular infection of a 73-year-old female with diabetes mellitus, four isolates were from external ocular infection from 27, 47, 48 and 64-years–old males and one of the isolates was from external ocular infection a 21-year–old female. All isolates were susceptible to linezolid and vancomycin. Of the 8 *S. aureus*, 5 isolates were methicillin-sensitive *Staphylococcus aureus* (MSSA) and were susceptible to amikacin, gentamicin, ciprofloxacin, clindamycin, erythromycin, trimethoprim/sulfamethoxazole, and tetracycline. The *pvl, mecA, ermA, ermB, ermC* and *msrA/B* genes were not detected in MSSA isolates. Three (37.5%) *S. aureus* isolates were resistant to cefoxitin and considered as MRSA. The isolates were not inducible clindamycin resistance. The *ermC* and *pvl* genes were only detected in a MRSA isolate obtained from external ocular infection of a 43-year-old male with diabetes mellitus. This isolate belonged to SCC*mec* IV and was resistant to trimethoprim/sulfamethoxazole, amikacin, gentamicin, ciprofloxacin, clindamycin, erythromycin, and tetracycline. Two MRSA isolates were from external ocular infection of 47 and 63-years-old males were not typeable with SCC*mec* typing method, were susceptible to amikacin, gentamicin, ciprofloxacin, clindamycin, erythromycin, tetracycline, and trimethoprim/sulfa-methoxazole.

## DISCUSSION

According to literature, *S. aureus* is the most important Gram-positive bacterium in ocular infections (1, 2). A wide range of resistance genes in *S. aureus* isolates has been reported from different samples in previous studies (1–3). Epidemiological investigations revealed that ocular infections by MRSA are increasing worldwide (14). Also, many studies in the ocular infection literature have shown that MRSA isolates are increasing in local settings (1, 2). As well, data suggested CA-MRSA isolates are the major threat in the ophthalmic settings and ocular infections (1, 2).

According to epidemiological evidence, prevalence of MRSA isolates in eye infections is 34% to 53% in different countries such as the United States, India and Taiwan (15, 16). The most important challenge in dealing with MRSA bacteria is to reduce the antibiotic choices in empirical therapy or prophylaxis because these isolates are commonly multi-drug resistant (MDR) (1). Diabetic patients are at a higher risk of getting infections than non-diabetics and ocular infection usually occurs in diabetic patients after eye surgery (2). In our study, all three MRSA bacteria were obtained from diabetic patients. Several studies, reported spread of MRSA isolates among ocular infections (1, 2, 16, 17). In a study in 2010 by Khan et al., MRSA isolates, belonging to SCC*mec* II and III, were reported in patients with ocular infections (18). MRSA isolates with SCC*mec* types I to III were considered as HA-MRSA and MRSA isolates with SCC*mec* type IV were considered as CA-MRSA (19). Detection of SCC*mec* IV and *pvl* genes in a MRSA isolate in our study revealed this isolate closely related to CA-MRSA (2, 3, 20). CA-MRSA isolates usually harbor panton-valentine leukocidin (PVL), therefore, ocular infections caused by CA-MRSA leads to more damage to the eye (16). In the present study, two MRSA isolates were not typeable in SCC*mec* typing. These results showed that other types of SCC*mec* may be spreading among MRSA isolates from ocular infections in our region.

In conclusion, MRSA isolates are commonly multi-drug resistant, therefore, antibacterial susceptibility test results should be considered in treatment of eye infections. On the other hand, CA-MRSA are usually PVL positive which increases eye damage, hence, genetic characteristics and molecular typing of MRSA isolates are very useful for detection and infection control.
